# (4*R*)-4-(Biphenyl-4-yl)-7-chloro-1,2,3,4-tetra­hydro­quinoline

**DOI:** 10.1107/S160053681103830X

**Published:** 2011-09-30

**Authors:** Thomas Theissmann, Michael Bolte

**Affiliations:** a Institute of Organic Chemistry, RWTH Aachen University, Landoltweg 1, 52074 Aachen, Germany; b Institut für Anorganische Chemie, J. W. Goethe-Universität Frankfurt, Max-von-Laue-Str. 7, 60438 Frankfurt/Main, Germany

## Abstract

The title compound, C_21_H_18_ClN, was synthesized by an enanti­oselective Brønsted acid-catalysed transfer hydrogenation reaction. The six-membered heterocycle adopts a half-chair conformation. It has the biphenyl residue in an axial position. The two rings of the biphenyl residue are almost coplanar [dihedral angle = 2.65 (9)°]. The crystal packing is stabilized by N—H⋯Cl hydrogen bonds, which connect the mol­ecules into chains running along the *a* axis.

## Related literature

For organocatalysed processes, see: Rueping, Sugiono & Schoepke (2010 ▶); Rueping, Dufour & Schoepke (2011 ▶). For Brønsted acid-catalysed transfer hydrogenations, see: Rueping *et al.* (2008 ▶); Rueping, Stoeckel *et al.* (2010 ▶). For the synthesis of the title compound, see: Rueping, Theissmann *et al.* (2011 ▶).
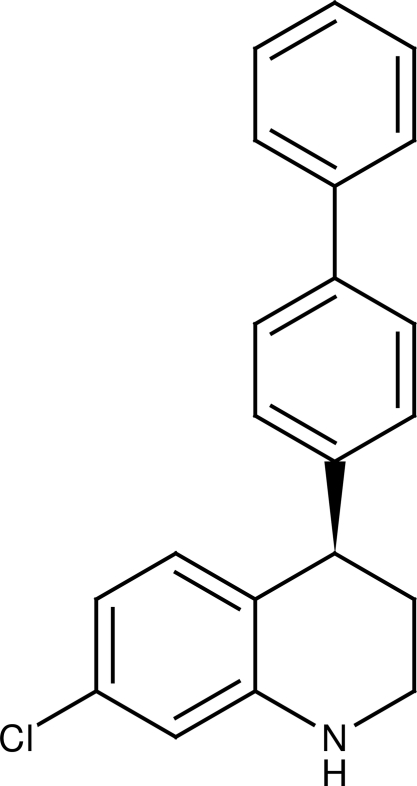


## Experimental

### 

#### Crystal data

C_21_H_18_ClN
*M* *_r_* = 319.81
Orthorhombic, 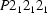

*a* = 5.5354 (4) Å
*b* = 8.0039 (4) Å
*c* = 35.8207 (17) Å
*V* = 1587.03 (16) Å^3^
*Z* = 4
Mo *K*α radiation
μ = 0.24 mm^−1^
*T* = 173 K
0.35 × 0.21 × 0.11 mm

#### Data collection

STOE IPDS II two-circle-diffractometer
Absorption correction: multi-scan (*MULABS*; Spek, 2009 ▶; Blessing, 1995 ▶) *T*_min_ = 0.921, *T*_max_ = 0.984
18042 measured reflections
3071 independent reflections
2867 reflections with *I* > 2σ(*I*)
*R*_int_ = 0.059

#### Refinement

*R*[*F* ^2^ > 2σ(*F* ^2^)] = 0.030
*wR*(*F* ^2^) = 0.079
*S* = 1.05
3071 reflections
213 parameters
H atoms treated by a mixture of independent and constrained refinement
Δρ_max_ = 0.21 e Å^−3^
Δρ_min_ = −0.18 e Å^−3^
Absolute structure: Flack (1983 ▶), 1240 Friedel pairs
Flack parameter: 0.01 (5)

### 

Data collection: *X-AREA* (Stoe & Cie, 2001 ▶); cell refinement: *X-AREA*; data reduction: *X-AREA*; program(s) used to solve structure: *SHELXS97* (Sheldrick, 2008 ▶); program(s) used to refine structure: *SHELXL97* (Sheldrick, 2008 ▶); molecular graphics: *XP* (Sheldrick, 2008 ▶); software used to prepare material for publication: *SHELXL97*.

## Supplementary Material

Crystal structure: contains datablock(s) I, global. DOI: 10.1107/S160053681103830X/lr2028sup1.cif

Structure factors: contains datablock(s) I. DOI: 10.1107/S160053681103830X/lr2028Isup2.hkl

Supplementary material file. DOI: 10.1107/S160053681103830X/lr2028Isup3.cml

Additional supplementary materials:  crystallographic information; 3D view; checkCIF report

## Figures and Tables

**Table 1:** Hydrogen-bond geometry (Å, °)

*D*—H⋯*A*	*D*—H	H⋯*A*	*D*⋯*A*	*D*—H⋯*A*
N1—H1⋯Cl1^i^	0.90 (3)	2.66 (3)	3.5466 (17)	171 (2)

Symmetry code: (i) 
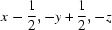
.

**Fig. 1.**
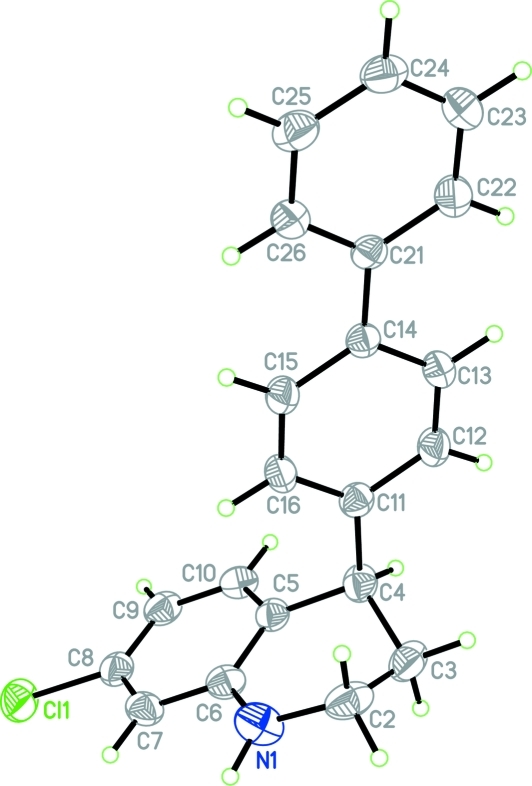
Perspective view of the title compound with the atom numbering; displacement ellipsoids are at the 50% probability level.

**Fig. 2.**
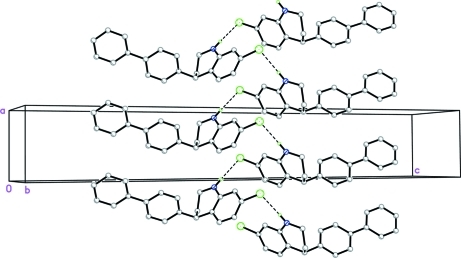
Packing diagram of the title compound. Hydrogen atoms bonded to C have been omitted for clarity. Hydrogen bonds are drawn as dashed lines.

## supplementary crystallographic information

### Comment

Tetrahydroquinolines are widely distributed in nature. Due to their importance as synthetic intermediates in the preparation of pharmaceuticals, agrochemicals, and in material science, considerable effort has been made to prepare these important molecules. Recently organocatalyzed processes have found widespread applications (Rueping, Sugiono & Schoepke, 2010; Rueping, Dufour & Schoepke, 2011). In particular Brønsted acid catalyzed transfer hydrogenations have been reported to provide a series of N-heterocyclic compounds with highest enantioselectivities (Rueping *et al.*, 2008; Rueping, Stoeckel *et al.*, 2010). The title compound was synthesized for the first time following this methodology (Rueping, Theissmann *et al.*, 2011) and colourless plates suitable for crystal structure determination were obtained.

The six-membered heterocycle in the title compound adopts a half chair conformation. It has the biphenyl residue in an axial position. The two rings of the biphenyl residue are almost coplanar [dihedral angle 2.65 (9)°]. The crystal packing is stabilized by N—H···Cl hydrogen bonds connecting the molecules to chains running along the *a* axis.

### Experimental

The title compound has been synthesized as described by Rueping, Theissmann *et al.* (2011).

### Refinement

All H atoms could be located by difference Fourier synthesis. Those bonded to C were refined with fixed individual displacement parameters [U(H) = 1.2 *U*_eq_(C)] using a riding model with C—H ranging from 0.95Å to 1.00 Å. The H atom bonded to N was freely refined.

### Crystal data

**Table:**

C_21_H_18_ClN	*F*(000) = 672
*M**_r_* = 319.81	*D*_x_ = 1.339 Mg m^−^^3^
Orthorhombic, *P*2_1_2_1_2_1_	Mo *K*α radiation, λ = 0.71073 Å
Hall symbol: P 2ac 2ab	Cell parameters from 18030 reflections
*a* = 5.5354 (4) Å	θ = 2.3–26.4°
*b* = 8.0039 (4) Å	µ = 0.24 mm^−^^1^
*c* = 35.8207 (17) Å	*T* = 173 K
*V* = 1587.03 (16) Å^3^	Plate, colourless
*Z* = 4	0.35 × 0.21 × 0.11 mm

### Data collection

**Table:**

STOE IPDS II two-circle-diffractometer	3071 independent reflections
Radiation source: fine-focus sealed tube	2867 reflections with *I* > 2σ(*I*)
graphite	*R*_int_ = 0.059
ω scans	θ_max_ = 25.9°, θ_min_ = 2.3°
Absorption correction: multi-scan (*MULABS*; Spek, 2009; Blessing, 1995)	*h* = −6→6
*T*_min_ = 0.921, *T*_max_ = 0.984	*k* = −9→9
18042 measured reflections	*l* = −43→44

### Refinement

**Table:**

Refinement on *F*^2^	Hydrogen site location: inferred from neighbouring sites
Least-squares matrix: full	H atoms treated by a mixture of independent and constrained refinement
*R*[*F*^2^ > 2σ(*F*^2^)] = 0.030	*w* = 1/[σ^2^(*F*_o_^2^) + (0.0482*P*)^2^ + 0.1208*P*] where *P* = (*F*_o_^2^ + 2*F*_c_^2^)/3
*wR*(*F*^2^) = 0.079	(Δ/σ)_max_ < 0.001
*S* = 1.05	Δρ_max_ = 0.21 e Å^−^^3^
3071 reflections	Δρ_min_ = −0.18 e Å^−^^3^
213 parameters	Extinction correction: *SHELXL*, Fc^*^=kFc[1+0.001xFc^2^λ^3^/sin(2θ)]^-1/4^
0 restraints	Extinction coefficient: 0.026 (2)
Primary atom site location: structure-invariant direct methods	Absolute structure: Flack (1983), 1240 Friedel pairs
Secondary atom site location: difference Fourier map	Flack parameter: 0.01 (5)

### Special details

**Table:**

Experimental. ;
Geometry. All e.s.d.'s (except the e.s.d. in the dihedral angle between two l.s. planes) are estimated using the full covariance matrix. The cell e.s.d.'s are taken into account individually in the estimation of e.s.d.'s in distances, angles and torsion angles; correlations between e.s.d.'s in cell parameters are only used when they are defined by crystal symmetry. An approximate (isotropic) treatment of cell e.s.d.'s is used for estimating e.s.d.'s involving l.s. planes.
Refinement. Refinement of *F*^2^ against ALL reflections. The weighted *R*-factor *wR* and goodness of fit *S* are based on *F*^2^, conventional *R*-factors *R* are based on *F*, with *F* set to zero for negative *F*^2^. The threshold expression of *F*^2^ > σ(*F*^2^) is used only for calculating *R*-factors(gt) *etc*. and is not relevant to the choice of reflections for refinement. *R*-factors based on *F*^2^ are statistically about twice as large as those based on *F*, and *R*- factors based on ALL data will be even larger.

### Fractional atomic coordinates and isotropic or equivalent isotropic displacement parameters (Å^2^)

**Table:**

	*x*	*y*	*z*	*U*_iso_*/*U*_eq_	
Cl1	0.19210 (10)	0.59051 (5)	0.005768 (11)	0.04965 (15)	
N1	0.1122 (3)	0.01504 (19)	0.06517 (4)	0.0428 (4)	
H1	−0.002 (5)	0.000 (3)	0.0477 (7)	0.059 (6)*	
C2	0.1862 (4)	−0.12356 (19)	0.08883 (4)	0.0407 (4)	
H2A	0.1373	−0.2302	0.0771	0.049*	
H2B	0.1034	−0.1151	0.1133	0.049*	
C3	0.4575 (4)	−0.12319 (19)	0.09483 (5)	0.0406 (4)	
H3A	0.5403	−0.1469	0.0709	0.049*	
H3B	0.5017	−0.2120	0.1128	0.049*	
C4	0.5406 (3)	0.04765 (18)	0.10990 (4)	0.0317 (3)	
H4	0.7213	0.0481	0.1100	0.038*	
C5	0.4573 (3)	0.18288 (17)	0.08315 (4)	0.0281 (3)	
C6	0.2442 (3)	0.15963 (19)	0.06229 (4)	0.0308 (3)	
C7	0.1654 (3)	0.28714 (19)	0.03814 (4)	0.0331 (3)	
H7	0.0211	0.2739	0.0241	0.040*	
C8	0.3000 (3)	0.43151 (18)	0.03507 (4)	0.0337 (3)	
C9	0.5160 (3)	0.45488 (18)	0.05393 (4)	0.0349 (3)	
H9	0.6092	0.5534	0.0506	0.042*	
C10	0.5906 (3)	0.32869 (19)	0.07781 (4)	0.0327 (3)	
H10	0.7381	0.3421	0.0910	0.039*	
C11	0.4564 (3)	0.07013 (17)	0.15017 (4)	0.0277 (3)	
C12	0.5861 (3)	−0.00906 (19)	0.17846 (4)	0.0319 (3)	
H12	0.7295	−0.0682	0.1723	0.038*	
C13	0.5108 (3)	−0.00350 (18)	0.21525 (4)	0.0311 (3)	
H13	0.6035	−0.0590	0.2338	0.037*	
C14	0.3006 (3)	0.08229 (16)	0.22576 (4)	0.0248 (3)	
C15	0.1743 (3)	0.16460 (19)	0.19743 (4)	0.0304 (3)	
H15	0.0325	0.2257	0.2035	0.037*	
C16	0.2518 (3)	0.15902 (19)	0.16041 (4)	0.0310 (3)	
H16	0.1628	0.2172	0.1418	0.037*	
C21	0.2160 (3)	0.08270 (16)	0.26536 (4)	0.0249 (3)	
C22	0.3477 (3)	0.00061 (19)	0.29322 (4)	0.0351 (4)	
H22	0.4934	−0.0551	0.2868	0.042*	
C23	0.2701 (3)	−0.0012 (2)	0.33005 (4)	0.0403 (4)	
H23	0.3632	−0.0577	0.3484	0.048*	
C24	0.0585 (3)	0.07846 (19)	0.34030 (4)	0.0357 (4)	
H24	0.0048	0.0762	0.3655	0.043*	
C25	−0.0739 (3)	0.1618 (2)	0.31320 (5)	0.0371 (4)	
H25	−0.2188	0.2178	0.3199	0.045*	
C26	0.0041 (3)	0.16382 (19)	0.27635 (4)	0.0321 (3)	
H26	−0.0888	0.2217	0.2582	0.039*	

### Atomic displacement parameters (Å^2^)

**Table:**

	*U*^11^	*U*^22^	*U*^33^	*U*^12^	*U*^13^	*U*^23^
Cl1	0.0746 (3)	0.0413 (2)	0.0330 (2)	0.0169 (2)	0.0022 (2)	0.00881 (16)
N1	0.0445 (8)	0.0444 (8)	0.0394 (8)	−0.0146 (7)	−0.0043 (7)	0.0067 (6)
C2	0.0615 (11)	0.0297 (7)	0.0309 (8)	−0.0111 (8)	0.0079 (8)	−0.0035 (6)
C3	0.0623 (11)	0.0287 (8)	0.0309 (8)	0.0079 (8)	0.0097 (8)	−0.0005 (6)
C4	0.0329 (7)	0.0325 (7)	0.0296 (7)	0.0056 (6)	0.0058 (6)	0.0022 (6)
C5	0.0316 (8)	0.0290 (7)	0.0237 (7)	0.0038 (6)	0.0040 (6)	−0.0007 (5)
C6	0.0341 (8)	0.0337 (7)	0.0246 (7)	−0.0020 (6)	0.0060 (6)	−0.0018 (5)
C7	0.0337 (8)	0.0420 (8)	0.0237 (7)	0.0033 (7)	0.0008 (6)	−0.0011 (6)
C8	0.0473 (9)	0.0304 (7)	0.0235 (7)	0.0083 (7)	0.0045 (7)	0.0002 (5)
C9	0.0461 (9)	0.0271 (7)	0.0315 (7)	−0.0031 (7)	0.0041 (7)	−0.0018 (6)
C10	0.0351 (8)	0.0345 (7)	0.0284 (7)	−0.0023 (6)	0.0025 (6)	−0.0044 (6)
C11	0.0301 (7)	0.0250 (6)	0.0280 (7)	−0.0008 (6)	0.0016 (6)	0.0003 (6)
C12	0.0287 (7)	0.0335 (8)	0.0336 (8)	0.0093 (6)	0.0012 (6)	0.0005 (6)
C13	0.0312 (7)	0.0327 (7)	0.0294 (7)	0.0073 (6)	−0.0046 (6)	0.0016 (6)
C14	0.0253 (6)	0.0214 (6)	0.0278 (6)	−0.0025 (6)	−0.0017 (6)	−0.0012 (5)
C15	0.0278 (7)	0.0329 (7)	0.0306 (7)	0.0083 (6)	0.0009 (6)	−0.0003 (6)
C16	0.0312 (8)	0.0334 (7)	0.0285 (7)	0.0079 (6)	−0.0021 (6)	0.0037 (6)
C21	0.0281 (7)	0.0201 (6)	0.0266 (6)	−0.0038 (6)	−0.0018 (5)	−0.0023 (5)
C22	0.0377 (8)	0.0345 (8)	0.0330 (8)	0.0069 (7)	0.0011 (7)	0.0025 (6)
C23	0.0523 (10)	0.0389 (8)	0.0296 (8)	0.0077 (7)	−0.0025 (7)	0.0058 (6)
C24	0.0491 (9)	0.0307 (7)	0.0273 (7)	−0.0037 (7)	0.0057 (7)	−0.0027 (6)
C25	0.0374 (9)	0.0396 (8)	0.0344 (8)	0.0023 (7)	0.0045 (7)	−0.0068 (7)
C26	0.0317 (8)	0.0345 (7)	0.0302 (7)	0.0036 (7)	−0.0027 (6)	−0.0013 (6)

### Geometric parameters (Å, °)

**Table:**

Cl1—C8	1.7545 (15)	C11—C16	1.387 (2)
N1—C6	1.372 (2)	C11—C12	1.394 (2)
N1—C2	1.455 (2)	C12—C13	1.383 (2)
N1—H1	0.90 (3)	C12—H12	0.9500
C2—C3	1.517 (3)	C13—C14	1.402 (2)
C2—H2A	0.9900	C13—H13	0.9500
C2—H2B	0.9900	C14—C15	1.397 (2)
C3—C4	1.540 (2)	C14—C21	1.4937 (19)
C3—H3A	0.9900	C15—C16	1.395 (2)
C3—H3B	0.9900	C15—H15	0.9500
C4—C5	1.5175 (19)	C16—H16	0.9500
C4—C11	1.5267 (19)	C21—C26	1.398 (2)
C4—H4	1.0000	C21—C22	1.399 (2)
C5—C10	1.394 (2)	C22—C23	1.388 (2)
C5—C6	1.409 (2)	C22—H22	0.9500
C6—C7	1.407 (2)	C23—C24	1.383 (2)
C7—C8	1.379 (2)	C23—H23	0.9500
C7—H7	0.9500	C24—C25	1.387 (2)
C8—C9	1.386 (2)	C24—H24	0.9500
C9—C10	1.386 (2)	C25—C26	1.389 (2)
C9—H9	0.9500	C25—H25	0.9500
C10—H10	0.9500	C26—H26	0.9500
			
C6—N1—C2	122.48 (15)	C5—C10—H10	118.8
C6—N1—H1	115.7 (15)	C16—C11—C12	117.50 (13)
C2—N1—H1	120.3 (15)	C16—C11—C4	124.03 (13)
N1—C2—C3	111.07 (14)	C12—C11—C4	118.41 (13)
N1—C2—H2A	109.4	C13—C12—C11	121.50 (13)
C3—C2—H2A	109.4	C13—C12—H12	119.2
N1—C2—H2B	109.4	C11—C12—H12	119.2
C3—C2—H2B	109.4	C12—C13—C14	121.44 (13)
H2A—C2—H2B	108.0	C12—C13—H13	119.3
C2—C3—C4	110.31 (13)	C14—C13—H13	119.3
C2—C3—H3A	109.6	C15—C14—C13	116.80 (13)
C4—C3—H3A	109.6	C15—C14—C21	122.13 (12)
C2—C3—H3B	109.6	C13—C14—C21	121.06 (12)
C4—C3—H3B	109.6	C16—C15—C14	121.43 (13)
H3A—C3—H3B	108.1	C16—C15—H15	119.3
C5—C4—C11	114.82 (12)	C14—C15—H15	119.3
C5—C4—C3	108.73 (13)	C11—C16—C15	121.28 (13)
C11—C4—C3	110.15 (12)	C11—C16—H16	119.4
C5—C4—H4	107.6	C15—C16—H16	119.4
C11—C4—H4	107.6	C26—C21—C22	117.03 (13)
C3—C4—H4	107.6	C26—C21—C14	122.11 (12)
C10—C5—C6	118.75 (14)	C22—C21—C14	120.86 (13)
C10—C5—C4	121.53 (14)	C23—C22—C21	121.44 (15)
C6—C5—C4	119.69 (13)	C23—C22—H22	119.3
N1—C6—C7	119.52 (15)	C21—C22—H22	119.3
N1—C6—C5	121.15 (14)	C24—C23—C22	120.65 (15)
C7—C6—C5	119.33 (14)	C24—C23—H23	119.7
C8—C7—C6	119.28 (14)	C22—C23—H23	119.7
C8—C7—H7	120.4	C23—C24—C25	118.89 (14)
C6—C7—H7	120.4	C23—C24—H24	120.6
C7—C8—C9	122.71 (14)	C25—C24—H24	120.6
C7—C8—Cl1	118.13 (13)	C24—C25—C26	120.44 (15)
C9—C8—Cl1	119.17 (12)	C24—C25—H25	119.8
C8—C9—C10	117.34 (14)	C26—C25—H25	119.8
C8—C9—H9	121.3	C25—C26—C21	121.54 (14)
C10—C9—H9	121.3	C25—C26—H26	119.2
C9—C10—C5	122.47 (15)	C21—C26—H26	119.2
C9—C10—H10	118.8		
			
C6—N1—C2—C3	−25.4 (2)	C5—C4—C11—C12	−157.97 (14)
N1—C2—C3—C4	54.08 (17)	C3—C4—C11—C12	78.90 (17)
C2—C3—C4—C5	−55.71 (17)	C16—C11—C12—C13	1.8 (2)
C2—C3—C4—C11	70.92 (17)	C4—C11—C12—C13	−175.56 (15)
C11—C4—C5—C10	88.01 (17)	C11—C12—C13—C14	−0.1 (2)
C3—C4—C5—C10	−148.10 (14)	C12—C13—C14—C15	−1.3 (2)
C11—C4—C5—C6	−93.87 (16)	C12—C13—C14—C21	177.88 (14)
C3—C4—C5—C6	30.02 (18)	C13—C14—C15—C16	1.1 (2)
C2—N1—C6—C7	178.40 (14)	C21—C14—C15—C16	−178.13 (13)
C2—N1—C6—C5	−1.7 (2)	C12—C11—C16—C15	−2.0 (2)
C10—C5—C6—N1	176.93 (14)	C4—C11—C16—C15	175.14 (14)
C4—C5—C6—N1	−1.2 (2)	C14—C15—C16—C11	0.6 (2)
C10—C5—C6—C7	−3.2 (2)	C15—C14—C21—C26	0.8 (2)
C4—C5—C6—C7	178.64 (13)	C13—C14—C21—C26	−178.40 (13)
N1—C6—C7—C8	−179.37 (14)	C15—C14—C21—C22	−179.32 (14)
C5—C6—C7—C8	0.7 (2)	C13—C14—C21—C22	1.5 (2)
C6—C7—C8—C9	2.3 (2)	C26—C21—C22—C23	0.5 (2)
C6—C7—C8—Cl1	−178.17 (11)	C14—C21—C22—C23	−179.43 (14)
C7—C8—C9—C10	−2.6 (2)	C21—C22—C23—C24	0.1 (2)
Cl1—C8—C9—C10	177.80 (11)	C22—C23—C24—C25	−0.6 (2)
C8—C9—C10—C5	0.0 (2)	C23—C24—C25—C26	0.5 (2)
C6—C5—C10—C9	2.8 (2)	C24—C25—C26—C21	0.1 (2)
C4—C5—C10—C9	−179.01 (14)	C22—C21—C26—C25	−0.6 (2)
C5—C4—C11—C16	24.9 (2)	C14—C21—C26—C25	179.31 (14)
C3—C4—C11—C16	−98.23 (17)		

### Hydrogen-bond geometry (Å, °)

**Table:**

*D*—H···*A*	*D*—H	H···*A*	*D*···*A*	*D*—H···*A*
N1—H1···Cl1^i^	0.90 (3)	2.66 (3)	3.5466 (17)	171 (2)

Symmetry codes: (i) *x*−1/2, −*y*+1/2, −*z*.
